# Comparison of Machine Learning Approaches to Improve Diagnosis of Optic Neuropathy Using Photopic Negative Response Measured Using a Handheld Device

**DOI:** 10.3389/fmed.2021.771713

**Published:** 2021-12-03

**Authors:** Tina Diao, Fareshta Kushzad, Megh D. Patel, Megha P. Bindiganavale, Munam Wasi, Mykel J. Kochenderfer, Heather E. Moss

**Affiliations:** ^1^Department of Management Science & Engineering, Stanford University, Stanford, CA, United States; ^2^Department of Ophthalmology, Stanford University, Palo Alto, CA, United States; ^3^Department of Aeronautics and Astronautics, Stanford University, Stanford, CA, United States; ^4^Department of Neurology and Neurological Sciences, Stanford University, Palo Alto, CA, United States

**Keywords:** photopic negative response (PhNR), electroretinogram (ERG), optic neuropathy, classification, machine learning, time series analysis

## Abstract

The photopic negative response of the full-field electroretinogram (ERG) is reduced in optic neuropathies. However, technical requirements for measurement and poor classification performance have limited widespread clinical application. Recent advances in hardware facilitate efficient clinic-based recording of the full-field ERG. Time series classification, a machine learning approach, may improve classification by using the entire ERG waveform as the input. In this study, full-field ERGs were recorded in 217 eyes (109 optic neuropathy and 108 controls) of 155 subjects. User-defined ERG features including photopic negative response were reduced in optic neuropathy eyes (*p* < 0.0005, generalized estimating equation models accounting for age). However, classification of optic neuropathy based on user-defined features was only fair with receiver operating characteristic area under the curve ranging between 0.62 and 0.68 and F1 score at the optimal cutoff ranging between 0.30 and 0.33. In comparison, machine learning classifiers using a variety of time series analysis approaches had F1 scores of 0.58–0.76 on a test data set. Time series classifications are promising for improving optic neuropathy diagnosis using ERG waveforms. Larger sample sizes will be important to refine the models.

## Introduction

Optic neuropathies cause visual impairment due to reduced function of the optic nerves, which carry the neurological signal generated by the photoreceptors and processed by the retina to the brain. In current clinical practice, detection of vision loss due to optic neuropathies is done by querying voluntary responses of patients to different visual stimuli, for example, through visual acuity testing or standard automated perimetry. Such psychophysical testing is subjective and can have significant fluctuations, which limits the reliability and undermines the accuracy of the evaluations. There is a need for more objective measures for detecting and monitoring visual dysfunction due to optic neuropathies.

Electroretinography is an objective measurement of the electrical discharge of the eye in response to light stimuli. The photopic negative response (PhNR) is the slow negative component of the photopic full-field electroretinogram (FF-ERG) that occurs after the b-wave. The PhNR relates to the retinal ganglion cells (RGCs) that form the optic nerve (1) and the amplitude of the PhNR is reduced in subjects with optic neuropathy (ON) including idiopathic intracranial hypertension (IIH) (2), glaucoma (3), optic nerve atrophy (4), and optic neuritis (5). The full-field stimulus PhNR offers advantages over an alternative electroretinographic measure of optic nerve function, the pattern ERG (PERG), because it does not require refraction or central fixation. PERG and PhNR performed similarly in detection of manifest glaucoma (6). In a study comparing IIH subjects with healthy controls, PhNR was impaired, while PERG was not, suggesting PhNR may be more sensitive (7). However, requirements for mydriasis, bench top stimulator and recording devices, and technical expertise to administer the test have limited further study of the PhNR as a diagnostic test in high volume clinical and research settings.

An integrated handheld ERG device that administers light stimulus protocols based on pupil size to allow non-mydriatic testing and records from skin electrodes is available commercially. This is more practical for widespread clinical use than traditional ERG setups (8). Though the skin electrodes reduce the amplitude of the signal (9), studies using this device have demonstrated correlation between PhNR amplitude and structural measures of the optic nerve in healthy adults (10) and people with glaucoma (11). Waveform processing approaches including utilization of ratios to normalize the PhNR amplitude to amplitudes of the b- and/or a-waves of the ERG (11) and detrending the baseline (12) have been shown to reduce variability in operator-defined variables. However, classification of clinical state on the basis of PhNR alone has been challenging and it has been deemed not ready for widespread use in optic neuropathies (13).

Analysis based on user-defined variables, even those that are normalized, fails to consider all the information in the ERG waveform and may contribute to poor classification performance. Consideration of all the points in the waveform increases dimensionality of the classification problem and may improve classification. Expanding analysis to consider how the points relate to each other as a time series further increases both the dimensionality and classification potential. Machine learning approaches to time series classification can be used to address this high-dimensional challenge. Specifically, supervised approaches can be used to generate models to classify patient diagnosis using the entire ERG waveform as the input. While machine learning has been broadly applied to image analysis in ophthalmology (14, 15), its application to ophthalmic electrophysiology including visual evoked potential (16–19), electro-oculography (20), PERG (21, 22), and the full-field ERG (23) has been limited. Brain electrophysiology [electroencephalography (EEG)] (24, 25) and cardiac electrophysiology (26, 27) have seen broader application with excellent results.

The objective of this study was to compare classifiers of the photopic full-field ERG, optimized for PhNR, as measured using a handheld non-mydriatic ERG device with skin electrodes, based on ability to differentiate ON from non-ON eyes in a neuro-ophthalmology practice.

## Materials and Methods

### Subjects

Adult subjects with bilateral, unilateral, or no ON were recruited from patients with outpatient appointments at the Byers Eye Institute at Stanford Neuro-ophthalmology Clinic. Each eye was assessed for inclusion either as an ON (ON+) eye or control (ON-) eye. A subject could contribute eyes to one or both the groups. All the ON (ON+) eyes had clinical evidence of ON such as optic nerve edema with visual acuity or peripheral vision impairment, optic nerve pallor with visual acuity or peripheral vision impairment, or structural thinning of ganglion cell layers on optical coherence tomography (OCT). No suspected cases (e.g., optic nerve drusen without other sign of optic nerve impairment) or resolved cases (e.g., treated and resolved papilledema with normal vision and OCT) were included in either group. Exclusion criteria included ophthalmic disease other than ON. Refractive error or mild cataract was permissible. An additional exclusion criterion for control eyes was neurological disease. Study of inclusion and exclusion criteria was based on medical record review by an attending neuro-ophthalmologist.

An additional group of control subjects without self-reported history of neurological or ophthalmic disease were recruited at the Spencer Center for Vision Research. All the subjects had baseline data collected. Those who had clinical follow-up during the study period were invited to have repeat measurements taken. This study was performed according to the tenets of the Declaration of Helsinki and was approved by the Stanford University Institutional Review Board. All the participants provided informed consent prior to data collection. Recruitment and data collection occurred from February 2017 to August 2018.

Age, gender, race/ethnicity, and nature of ON were extracted from the medical record for subjects recruited from the clinic. Age, gender, and race/ethnicity were self-reported by subjects recruited from the research center. Eyes of the ON subjects were classified as ON or control-fellow eye. ON eyes were classified as acute, if the optic nerve disease started less than 3 months prior to enrollment or chronic. Eyes of the control subjects were classified as control-patient, if they were recruited from the clinic (i.e., had no afferent neuro-ophthalmic disease) or control-healthy, if they had no known ophthalmic or neurological disease.

### Visual Function and Ancillary Testing

Best corrected Snellen visual acuity was extracted from the clinical record for subjects recruited from the clinic. Distance visual acuity with habitual correction was measured for control-healthy subjects. Snellen visual acuity was converted to the logarithm of the minimum angle of resolution (logMAR) for the purposes of analysis. Count fingers, hand motions, and no light perception were assigned logMAR 2, 3, and 6, respectively.

Ancillary ophthalmic testing was included, if it was collected as a part of clinical care. For subjects who had OCT cube scans of the macula (512 × 128) and/or the optic disk (200 × 200) (Cirrus; Carl Zeiss Meditech Incorporation, Jena, Thuringia, Germany, UK), average retinal nerve fiber layer (RNFL) and ganglion cell layer plus inner plexiform layer (GCL + IPL) thickness, as calculated by Zeiss software, were recorded. OCT measures were included in analysis, if signal strength ≥ 7.

For subjects who had visual field testing (24-2 or 30-2 SITA-FAST, Humphrey Field Analyzer; Carl Zeiss Meditech Incorporation, Jena, Thuringia, Germany, UK), the Humphrey Visual Field mean deviation (HVF-MD) in decibels was recorded. HVF was included in analysis, if fixation losses ≤ 6, false negatives <20%, and false positives <20%.

### Electroretinography

Stimulation and recording were performed in an interior examination room with the lights off. Subjects were seated without mydriasis. Following cleaning of the skin below the lower lids with alcohol swabs, adhesive skin electrodes were placed 2 mm below the lower lid of each eye extending laterally with the medial end aligned with the center of the eye. The photopic ERG was recorded sequentially in both the eyes using a portable commercial device (RETeval, LKC Technologies Incorporation, Gaithersburg, Maryland, USA). Full-field stimulation red (621 nm) flashes (58 Tds) were delivered at 3.4 Hz over a blue (470 nm, 380 Td) background to each eye. Software within the device applied a 1-Hz high-pass filter and 100 Hz low-pass filter, removed outliers, used a trimmed mean to combine the results from individual flashes, and applied wavelet-based denoising to generate an ERG waveform for each recording. A total of 300 flashes were delivered in each eye over two or three recordings. For the first 23 subjects, these were divided into one 100 flash recording and one 200 flash recording. For the remaining subjects, three 100 flash recordings were completed. Testing sessions lasted under 10 min per subject.

Averaged ERG waveforms for each recording (220 ms with 430 data points) with device software filtering and outlier removal were extracted from RFF files generated by the device and were used as input for analyses involving user-defined features and the full-time series.

### Analysis of User-Defined ERG Features

A custom script (MATLAB 2018a, MathWorks, Incorporation, Natick, Massachusetts, USA) was used to process the ERG waveforms for each recording. The input waveforms for MATLAB were the averaged, filtered waveform with outliers removed as generated by the RETeval device software. The linear trend was removed using the *detrend* function to account for steady upward or downward drifts in many of the recordings. The waveforms for each session were reviewed. Any outliers or those without a defined b-wave peak were excluded.

The following values were extracted from the detrended waveform for each included trial. The baseline value was calculated by averaging all the data points from the start of the recording to the time that the flash was administered (100 ms). The b-wave peak was defined at the maximum potential. The a-wave trough was defined at the minimum potential between the time the flash was administered and the time of the b-wave peak. The late negative response trough was defined in two ways in different analyses. First, it was defined at 72 ms after the stimulus (28). Second, it was defined at the minimum potential in a ± 10 ms window centered at 72 ms after the flash (29).

The a-wave amplitude (a_amp_) was calculated as the potential difference between the a-wave trough and baseline potential, while the b-wave amplitude (b_amp_) was calculated as the potential difference between the a-wave trough and the b-wave peak potential. PhNR_72_ amplitude was calculated as the difference between baseline potential and the potential at 72 ms. PhNR_min_ was calculated as the difference between baseline potential and the mean of 11 consecutive points (~5.62 ms) centered at the late negative response trough as done in previous studies (2, 7, 30). To account for waveform variability, the P-ratio $\left(-\frac{PhNR}{b_{amp}}\right)$ and the W-ratio $\left(\frac{b_{amp}-PhNR}{b_{amp}-a_{amp}}\right)$ were calculated (6, 28).

Each ERG feature (PhNR_72_, PhNR_min_, P-ratio, and W-ratio) was modeled as a function of ON status (ON+, ON-) using linear generalized estimating equations (GEEs) accounting for intrasubject correlation. ERG features were compared between different sources of control eyes (healthy, patient, and fellow) using GEE models. Linear GEE models were also used to model each ERG feature as a function of structural and functional measures of the optic nerve including RNFL thickness, GCL + IPL thickness, and HVF-MD.

Using one eye from each subject (right eye unless the subject only contributed a left eye), a receiver operating curve analysis was performed. The Youden index [maximum of (sensitivity + specificity −1)] was used to select the optimal cutoff point at which to calculate sensitivity and specificity for comparison with the time series analysis. Area under the curve was calculated for subset of ON eyes with visual field MD < −5 dB (i.e., severe ON) and controls. For comparison, area under the curve was calculated for device calculated features (i.e. PhNR, P-ratio, W-ratio reported by the device software prior to custom MATLAB analysis). Analysis was done using the SPSS software (version 26; IBM SPSS Statistics, IBM Corporation, Chicago, Illinois, USA).

### Time Series Analysis of ERG

**Figure 1:**
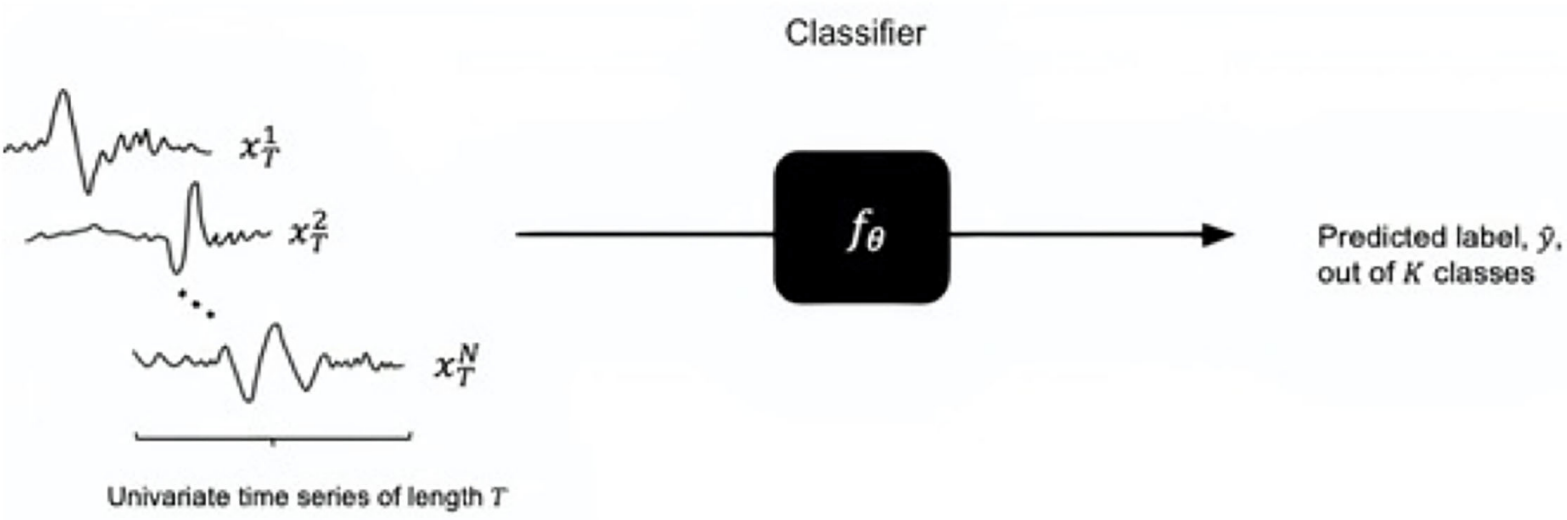
Time series classification task (for univariate time series): a trained classifier with parameters takes the input of time series of length T and outputs a predicted class out of K classes. The input size is denoted by N.

Time series classification (TSC) involves using time-ordered discrete attributes to make predictions of a class. Let X be the time series space and Y be the label space. Let (x, y) denote a labeled example, in which y is the label for x. The goal is to train a classifier *f*_θ_: X → Y such that *l[f*_θ_ *(x; y)]* is minimized for an objective function *l* and θ is the set of parameters associated with the classifier model (Figure 1). For the diagnostic task of ON, the input *x* is univariate time series of length *T* (i.e. the ERG waveform) and the output labels *y* = (−1, 1), namely *ON*+ and *ON-*, are binary (K = 2).

Classifiers were selected for comparison based on their benchmarked performances on other datasets in the machine learning literature (31). The classifiers we compared are nearest neighbor dynamic time warping (NN DTW), linear support vector machine (SVM linear), support vector machine with a radial basis function kernel (KBF kernel SVM), random forest classifier (RF), gradient boosted (GB) classifier, time series forest (TSF), and long-short term memory (LSTM) networks, a form of recurrent neural networks in deep learning, each of which is described in more detail below. The objective functions (*l)* used were DTW distance, hinge loss function, the Gini index, binomial deviance, and cross-entropy loss functions.

#### Nearest Neighbor Dynamic Time Warping

A label for an example is predicted based on the closest distance between the example *x*_1_ and another data series *x*_2_, its nearest neighbor (NN). DTW denotes the type of distance measure to be minimized between the two data series, where *d*_*DTW*_(*x*_1_, *x*_2_) is commonly the Euclidean distance between *x*_1*i*_ and *x*_2*j*_ for time indices *i, j*∈{1, ...*T*} with an optimal path along a sequence *w*(*i, j*) (32).

#### Linear Support Vector Machine

A linear support vector machine uses a linear classifier *f*_θ_ such that for an example (*x*_*k*_,*y*_*k*_) we have the prediction of *y*_*k*_ be (*f*_*x*_*k*__; θ) = θ*x*_*k*_, and we minimize a hinge loss, $l_{k}=C\cdot \max\left(0,\;1-y_{k}\theta^{T}x_{k}\right)+R\left(\theta \right)$, between the prediction and label. The *C* is a hyperparameter and *R*(θ) is the regularization penalty, commonly the *L*_2_ norm.

#### Linear Support Vector Machine With a Radial Basis Function Kernel

An RBF kernel SVM classifier is suitable for non-linear datasets (33). The KBF kernel for a pair of data series *x*_1_ and another data series *x*_2_ is *exp* (- γ||*x*_1_ - *x*_2_ ||^2^), where γ is a hyperparameter and ||*x*_1_ - *x*_2_ || is the Euclidean distance.

#### Random Forest

Random forest is an ensemble method that averages the predictions from a number of de-correlated decision trees (34). It is a non-linear classifier from construction of linear boundaries per tree node and reducing the node impurity. A common form of node impurity for a binary classification task is the Gini index, defined by 2*p* (1 - *p*) where *p* is the probability of the second class (35).

#### Gradient Boosting

Gradient boosting is another ensemble method that minimizes the loss function by introducing a tree with a prediction as close as possible to the negative gradient (36). The loss function for the binary classification task is binomial deviance, log [1+ exp (- 2 ·*y* ·*f(x)*)] for an example (*x, y*) and gradient boosted classifier *f* .

#### Time Series Forest

Time series forest is a modification of the random forest classifier for time series (37). It samples a set of random intervals and extracts mean, SD, and slope per interval to train the time series trees, reducing cross-entropy loss -[*y* log *p* + (1 - *y*) log (1-*p*)], where *p* is the probability of the second class.

#### Long-Short Term Memory Networks

The recurrent neural network architecture incorporates temporal dynamics by allowing information to be passed from one step of the network to the next (38). LSTMs (39) are widely used to address the vanishing gradient problem of recurrent neural networks (40). The loss function to minimize is the cross-entropy loss.

#### Training, Validation, and Testing of Machine Learning Models

Electroretinogram waveforms for all the trials (baseline, follow up) of included eyes were included in the time series analyses. The waveforms were split into training, validation, and testing sets. The split was done to ensure: (a) both the eyes of each records of the subject appeared only in one of the three sets and (b) the distributions of diagnostic outcomes were balanced in each set. For each of the classifiers studied, training was performed on the training set. The validation set was then used to tune the hyperparameters and select the best models for testing on the test set. The results of the test set classification were categorized as true positive (TP) (eye ON+, classifier ON+), true negative (TN) (eye ON-, classifier ON-), false negative (FN) (eye ON+, classifier ON-), and false positive (FP) (eye ON-, classifier ON+). The classifiers were compared on the basis of precision [TN/(TN + FP), equivalent to sensitivity], recall [TP/(TP + FN), equivalent to specificity], accuracy [(TP + TN)/(TP + TN + FP + FN)], and F1 score [precision × recall/(precision + recall)]. Experiments were performed in Python 3.8.6 and the main packages used were sktime, scikit-learn, and pytorch.

## Results

A total of 155 subjects were screened and 119 (63, 53% female, age 45.6 ± 17.5 years) had one or two eyes meeting inclusion criteria. Subjects were of diverse race and ethnicity with white non-Hispanic (68, 57%), Asian (22, 19%), and white-Hispanic (16, 13%) being the most prevalent.

A total of 217 eyes were included, of which 108 were control eyes (24 control-fellow eyes, 20 control-patient eyes, and 64 healthy-control eyes) and 109 were ON eyes (33 acute, 76 chronic). Diagnoses were papilledema (19 total; 15, idiopathic intracranial hypertension 4 other high intracranial pressure), compressive (17 total; 12 chiasm, 3 intracranial, 2 orbit), non arteritic anterior ischemic optic neuropathy (16), atrophy (15), optic neuritis (14), glaucoma (4), optic nerve head edema (4), toxic (4), tract (4), inflammatory (3), Leber hereditary ON (2), dominant optic atrophy with OPA1 mutation (2), optic disk drusen (2), and one each infection, orbit inflammation and trauma. ON eyes had worse visual acuity (VA) and more impaired peripheral vision. Acute ON eyes had thicker RNFL than control eyes, while chronic ON eyes had thinner RNFL and GCP + IPL (Table 1).

**Table 1:** Unadjusted comparison between eyes with and without optic neuropathy.

	**Optic neuropathy eyes (*n =* 109 unless noted)**	**Control eyes (*n =* 108 unless noted)**	**Comparison p (GEE)**
Age in years (mean ± SD)	48.9 ± 17.2	40.0 ± 15.3	*p =* 0.002
Female gender (n)	52	64	*p =* 0.162
VA in logMAR (median, range)	0.18 (−0.3, 6)	0 (−0.2, 1)	*p < * 0.0005
HVF-MD in dB (mean +/– SD)	−10.6 ± 10.1 (*n =* 77)	−1.1 ± 2.19 (*n =* 28)	*p < * 0.0005
OCT RNFL in μm (mean +/– SD)			
Acute	182 ± 104 (*n =* 21)		
Chronic	69 ± 14 (*n =* 63)	97 ± 9 (*n =* 31)	*p < * 0.0005
OCT GCL+IPL in μm (mean +/– SD)			
Acute	64 ± 17 (*n =* 28)		
Chronic	60 ± 10 (*n =* 67)	81 ± 11 (*n =* 29)	*p < * 0.0005
PhNR_min_in μV	−2.8 ± 1.5	−3.7 ± 1.8	*p < * 0.0005
PhNR_72_in μV	−1.4 ± 1.7	−2.0 ± 2.4	*p =* 0.06
P-ratio	0.12 ± 0.10	0.17 ± 0.14	*p =* 0.02
W-ratio	0.97 ± 0.15	1.01 ± 0.13	*p =* 0.025

*GEE, generalized estimating equation; VA, visual acuity; HVF-MD, Humphrey visual field mean deviation; OCT, optical coherence tomography; RNFL, retinal nerve fiber layer; GCL, ganglion cell layer; IPL, inner plexiform layer; PhNR, photopic negative response*.

### User-Defined ERG Features

Waveforms were not available for three subjects (five eyes). For these, PhNR features as measured by the commercial software included with the acquisition device were used. PhNR, P-ratio, and W-ratio had lower magnitudes in ON than control eyes (Table 1) and this persisted when accounting for age (*p* < 0.0005, 0.009, 0.031). Among control eyes, ERG features did not differ by source of control (fellow eye vs. other controls; healthy eyes vs. other controls; *p* = 0.38–92, GEE).

For subjects with available structural and other functional measures of ON, HVF-MD was linearly related to PhNR_min_ (*p* = 0.002, GEE), but the relationships with PhNR_72_ (*p* = 0.09, GEE), P-ratio (*p* = 0.10, GEE), and W-ratio (*p* = 0.09, GEE) did not meet statistical significance. Among control and chronic ON eyes with available OCT, RNFL was linearly related to PhNR_min_ (*p* = 0.004, GEE), but not PhNR_72_ (*p* = 0.58, GEE), P-ratio (*p* = 0.37, GEE), or W-ratio. GCL + IPL was related to P-ratio (*p* = 0.009, GEE), but not PhNR_min_ (*p* = 0.45, GEE), PhNR_72_ (*p* = 0.06, GEE), or W-ratio (*p* = 0.34, GEE).

**Figure 2:**
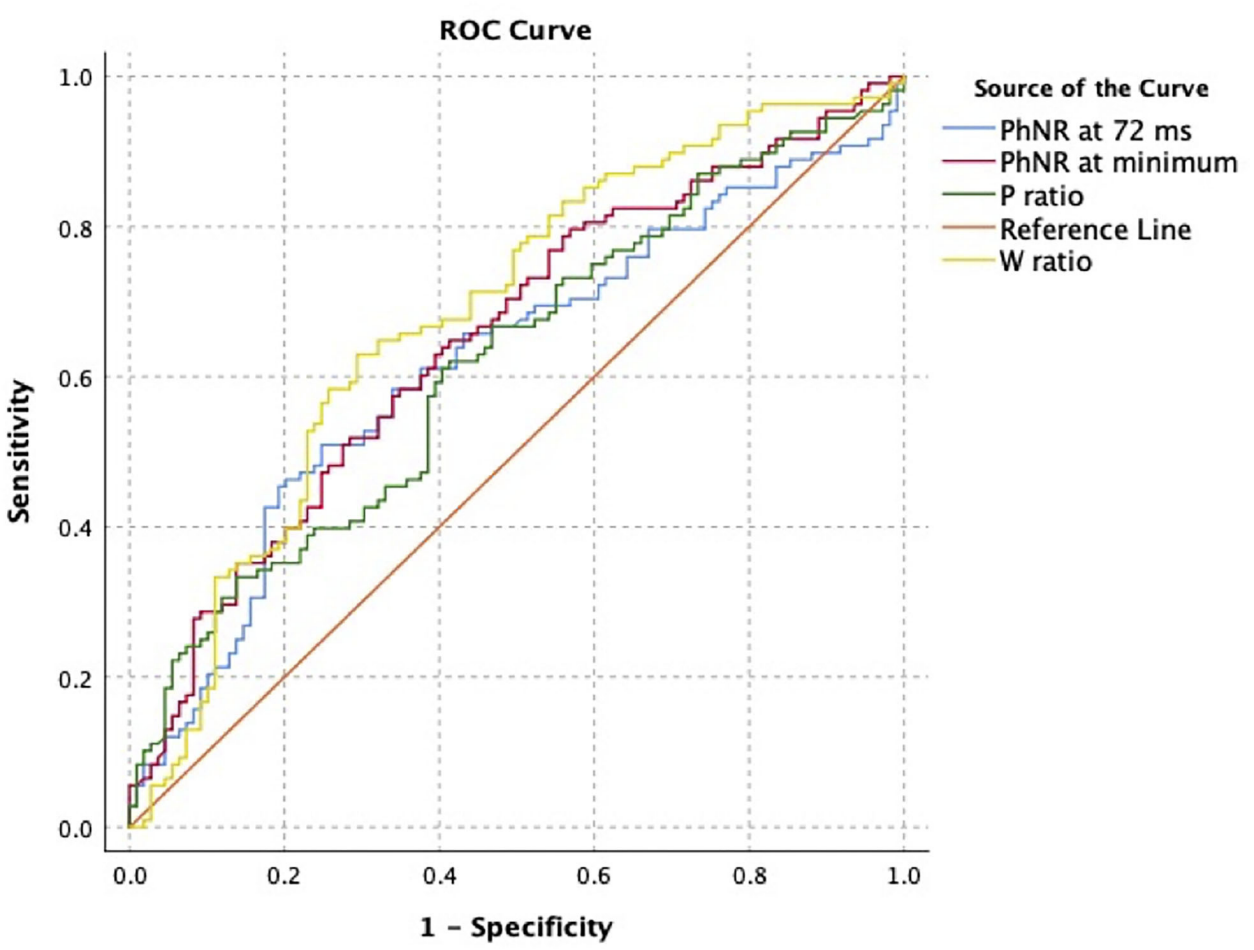
Receiver operating characteristic analysis for classification of optic neuropathy status using user-defined electroretinogram (ERG) features. Curve was constructed using one eye per subject.

In analysis of classification potential using one eye per subject (63 ON+, 56 ON-), receiver operating curve analysis showed fair classification potential (Table 2, Figure 2). At the optimal cutoff as selected using the Youden index, PhNR_72_ had the best sensitivity (0.75), while W-ratio had the best specificity (0.71). Areas under the curves were similar when analysis was restricted to eyes with severe ON (AUC 0.64–0.68). Areas under the curves were similar for device calculated parameters (AUC 0.64–0.69).

**Table 2:** Receiver operating curve analysis for classification of optic neuropathy using user-defined ERG features in all the subjects.

**Feature**	**Area under curve**	**Youden index**	Sensitivity^*^	Specificity^*^	F1 score^*^
PhNR_min_	0.65	0.23	0.61	0.63	0.31
PhNR_72_	0.62	0.26	0.75	0.51	0.30
P-ratio	0.62	0.20	0.62	0.59	0.30
W-ratio	0.68	0.34	0.63	0.71	0.33

*Analysis included one eye per subject*.

* *values for optimal cutoff as determined using the Youden index. PhNR, photopic negative response; ERG, electroretinogram*.

### Time Series Analysis

For the included eyes, there were a total of 791 available waveforms for baseline and follow-up visits in 115 unique subjects (3 subjects for whom ERG waveforms were not available did not contribute to this analysis). The prevalence rate for ON was 0.57. The numbers of waveforms in training, tuning, and testing sets were 258, 161, and 172, respectively. The numbers of unique subjects in each set were 61, 21, and 33, respectively.

The most important parameters and classification results for the testing set are shown in Table 3. The highest precision (0.74), accuracy (0.74), and F1 score were achieved by TSF with 100 estimators used. The highest recall (0.86) was achieved by the RBF Kernel SVM with a regularization constant of 1.5.

**Table 3:** Classifier objectives parameters used in the best performing model on the testing set and the results from the testing set.

**Classifier**	**Objective to minimize**	**Important parameters used**	**Precision (for “ON”) **~**sensitivity**	**Recall (for “ON”) **~**specificity**	**Accuracy**	**F1 Score**
1-NN DTW	DTW distance	-	0.64	0.72	0.65	0.68
SVM Linear	Hinge loss	-	0.63	0.79	0.63	0.70
RBF Kernel SVM	RBF distance	Regularization constant = 1.5, Gamma = 1 / (n_features × var(X))	0.66	**0.86**	0.69	0.74
RF	Gini index	N_estimators = 200	0.73	0.77	0.73	0.75
GB	Binomial deviance	N_estimators = 100	0.70	0.76	0.70	0.73
TSF	Cross entropy	N_estimators = 100	**0.74**	0.78	**0.74**	**0.76**
LSTMs	Cross entropy	N_layers = 3, Batch_size = 6, Dropout = 0.6, Hidden dims = 16, Optimizer = Adam, Learning_rate = 0.001 N_epochs = 100	0.46	0.78	0.68	0.58

*Bold indicates best results. NN DTW, nearest neighbor dynamic time warping; SVM, support vector machine; RBF Kernel SVM, support vector machine with a radial basis function kernel; RF, random forest; GB, gradient boosting; TSF, time series forest; LSTMs, long-short term memory*.

## Discussion

Optic neuropathies are important to diagnose because they impair vision and often reflect underlying neurological or neurosurgical disease. This study investigates the utility of the full-field ERG, analyzed based on user-defined features or time series analysis, to classify eyes as having ON. Attention was given to having a clinically feasible protocol and a representative clinical sample. Specifically, the protocol including lack of mydriasis, using skin electrodes and a portable stimulating and recording device was implemented in a clinic room in less than 10 min per subject. The sample included ON eyes of different etiologies ranging in severity and control eyes of three types (fellow eyes from subjects with unilateral ON, those with non-afferent reasons for visiting the neuro-ophthalmology clinic, and healthy controls). These protocol and sample features increase the translational potential of the findings to clinical practice.

Consistent with prior reports, we found a statistically significant difference in user-defined ERG measures including PhNR trough and at 72 ms, P-ratio, and W-ratio between eyes with and without ON. Also, in line with prior reports, correlation is found between user-defined ERG features with some markers of ON including function and structure. However, the classification ability based on user-defined features is fair at best within our data. This is despite using customized waveform analysis, excluding outlier tracings, and including all the available data in classification analysis. This is likely an overestimate of performance as we present only training results. This is because we did not have a sufficient sample size to divide the sample into training and testing sets for the user-defined feature analysis. It is likely that an independent test set would have worse performance.

Using a time series analysis that makes use of all the information in the waveform, we achieved better classification for an independent test data set than was obtained in training based on user-defined features. In general, the ensemble methods (RF, GB, and TSF) produced above 0.7 for all the metrics. The higher performances are corroborated by Bagnall et al. (31). The LSTMs did not achieve a high accuracy, despite a high recall score. Deep learning models have not been widely considered for time series classification tasks, despite their popularity in other application areas (41). In particular, recurrent neural networks are difficult to train and may suffer from the aforementioned vanishing gradient problem, which is addressed by LSTMs. Our results show promise in developing such neural networks for high sensitivity of disease detection.

The main limitation of this study is the data set size. This limited our ability to do split training and testing data sets for user-defined features and to pursue stratified analysis (e.g., based on ON severity or covariates). A larger sample size would also likely to improve tuning of time series models. For example, classification of ECG signals for diagnosis of heart disease has reported better performances (>95%) using the same metrics with machine learning algorithms using a 4,000-sample MIT-BIH database (https://physionet.org/physiobank/database/html/mitdbdir/intro.htm) (42, 43). The nature of the ERG protocol also introduced limitations. Specifically, the amplitude of the signal from skin electrodes is lower signal than traditional DTL or other corneal electrodes and recording in a dim room without light adaptation may have increased variability (44).

In conclusion, a portable ERG device using a non-mydriatic stimulation protocol and skin electrodes in subjects attending a neuro-ophthalmology clinic with and without ON and control subjects measured PhNR amplitude decrease in eyes with ON vs. control eyes. While classification of ON status based on user-defined features was fair, time series classification models developed using machine learning techniques demonstrated better classification performance. Portable non-mydriatic ERG recorded using skin electrodes and time series classification analysis may have application to using the full-field ERG as a bedside diagnostic test for ON.

## Data Availability Statement

De-identified data will be made available upon reasonable request to the corresponding author by any qualified researcher.

## Ethics Statement

The studies involving human participants were reviewed and approved by Stanford University Institutional Review Board. The patients/participants provided their written informed consent to participate in this study.

## Author Contributions

HM and MK contributed to the conception. FK, MB, and MW contributed to the data collection. TD, FK, MP, and MB contributed to the data analysis and interpretation. TD, FK, MP, and HM contributed to the drafting of the article. HM and MK contributed to the critical revision of the article.

## Funding

This study was supported by grants of the National Institutes of Health (K23 EY 024345 and P30 EY 026877) and an unrestricted grant from the Research to Prevent Blindness to the Stanford Department of Ophthalmology. The funding agencies had no influence over the design or interpretation of this study.

## Conflict of Interest

The RetEVAL device was provided for research use by LKC Technologies Inc. The company had no influence over the design or interpretation of this study. The authors declare that the research was conducted in the absence of any commercial or financial relationships that could be construed as a potential conflict of interest.

## Publisher's Note

All claims expressed in this article are solely those of the authors and do not necessarily represent those of their affiliated organizations, or those of the publisher, the editors and the reviewers. Any product that may be evaluated in this article, or claim that may be made by its manufacturer, is not guaranteed or endorsed by the publisher.
